# Ethylene Response Factor109 Attunes Immunity, Photosynthesis, and Iron Homeostasis in Arabidopsis Leaves

**DOI:** 10.3389/fpls.2022.841366

**Published:** 2022-03-02

**Authors:** Chiu-Ling Yang, Yu-Ting Huang, Wolfgang Schmidt, Patricia Klein, Ming-Tsair Chan, I-Chun Pan

**Affiliations:** ^1^Department of Horticulture, National Chung-Hsing University, Taichung City, Taiwan; ^2^Institute of Plant and Microbial Biology, Academia Sinica, Taipei, Taiwan; ^3^Department of Horticultural Sciences, Texas A&M University, College Station, TX, United States; ^4^Biotechnology Center in Southern Taiwan, Academia Sinica, Tainan, Taiwan

**Keywords:** *ERF109*, *RRTF1*, iron deficiency, immunity, photosynthesis, transcriptome

## Abstract

Iron (Fe) is an essential micronutrient element for all organisms including plants. Chlorosis of young leaves is a common symptom of Fe deficiency, reducing the efficiency of photosynthesis, and, ultimately, crop yield. Previous research revealed strong responsiveness of the putative key transcription factor *ERF109* to the Fe regime. To elucidate the possible role of *ERF109* in leaf Fe homeostasis and photosynthesis, we subjected *Arabidopsis thaliana erf109* knockout lines and Col-0 wild-type plants to transcriptome profiling *via* RNA-seq. The transcriptome profile of Fe-sufficient *erf109* leaves showed a 71% overlap with Fe-deficient Col-0 plants. On the other hand, genes that were differentially expressed between Fe-deficient and Fe-sufficient Col-0 plants remained unchanged in *erf109* plants under conditions of Fe deficiency. Mutations in *ERF109* increased the expression of the clade Ib bHLH proteins *bHLH38*, *bHLH39*, *bHLH101*, the nicotianamine synthase *NAS4*, and the Fe storage gene *FER1*. Moreover, mutations in *ERF109* led to significant down-regulation of defense genes, including *CML37*, *WRKY40*, *ERF13*, and *EXO70B2*. Leaves of *erf109* exhibited increased Fe levels under both Fe-sufficient and Fe-deficient conditions. Reduced Fv/Fm and Soil Plant Analysis Development (SPAD) values in *erf109* lines under Fe deficiency indicate curtailed ability of photosynthesis relative to the wild-type. Our findings suggest that *ERF109* is a negative regulator of the leaf response to Fe deficiency. It further appears that the function of *ERF109* in the Fe response is critical for regulating pathogen defense and photosynthetic efficiency. Taken together, our study reveals a novel function of *ERF109* and provides a systematic perspective on the intertwining of the immunity regulatory network and cellular Fe homeostasis.

## Introduction

By virtue of its ability to change valency, iron (Fe) is a critical component of photosynthesis and respiratory electron transport, a constituent of Fe-sulfur clusters, and a cofactor of a multitude of vital redox enzymes. Owing to the chemical characteristic of Fe, free Fe ions in plant cells are highly redox active and can react with H_2_O_2_ to produce the reactive hydroxyl (⋅OH) radical in the so-called Fenton reaction, which can cause oxidative stress and cell damage when produced in excess (Floyd and Lewis, 1983; Baker and Gebicki, 1986). The most significant symptom of Fe deficiency in plants is interveinal chlorosis of young leaves. In Fe-deficient plants, compromised chlorophyll production reduces the efficiency of photosynthesis and causes a decrease in fruit yield and quality (Àlvarez-Fernàndez et al., 2006; Rombolà and Tagliavini, 2006). Insufficient Fe supply decreases the level of the electron donor cytochrome c6 and was shown to partially block the electron transfer between PSII and PSI in the marine diatom *Phaeodactylum tricornutum* (Roncel et al., 2016). Similar to Fe deficiency, high levels of light irradiation cause oxidative stress in plant cells (Erickson et al., 2015). When the light intensity exceeds the photosynthetic capacity of the plant, excessive energy can induce light inhibition of photosynthesis and cause the formation of excessive ROS species, resulting in leaf cell death (Karpiński et al., 2013). Systemic Acquired Acclimation (SAA) is induced by the exposure of leaves to high light stress, which triggers systemic signaling and preacclimation of shaded leaves (Rossel et al., 2007).

*ETHYLENE-RESPONSIVE TRANSCRIPTION FACTOR109* (*ERF109*), also described as *REDOX-RESPONSIVE TRANSCRIPTION FACTOR1* (*RRTF1*), is a member of the ERF/AP2 transcription factor family that is involved in a multitude of abiotic and biotic stresses, such as salt (Bahieldin et al., 2016, 2018; Soliman and Meyer, 2019), high light (Vogel et al., 2014; Carmody et al., 2016; Huang et al., 2017), and oxidative stress (Khandelwal et al., 2008; Matsuo et al., 2015; Pospisil, 2016; Kong et al., 2018), infection with *Alternaria brassicae* (Vahabi et al., 2018), as well as in essential processes such as hormone signaling and root stem cell maintenance (Cai et al., 2014; Kong et al., 2018; Vahabi et al., 2018). It was confirmed that high light stress-induced chloroplast singlet oxygen stress is the beginning of a systemic domestication reaction, transmitting a signal to the cell nucleus that regulates *ERF109* expression, and, subsequently, generates signal fluctuations between cells through the plasma membrane protein *RBOHD*/*F* (Carmody et al., 2016). Hence, the availability of Fe and the Fe nutritional status of the plant are important factors for light responsiveness. Furthermore, chloroplast retrograde and ethylene signaling were shown to be connected with Fe homeostasis (Balparda et al., 2020). Here, we attempt to validate the supposition that *ERF109* plays a critical role in the coordination of Fe deficiency, light signaling, and pathogen defense. Transcriptome analysis revealed that lines harboring defects in *ERF109* exhibited a similar pattern of a constitutively expressed subset of genes associated with the immune network, resembling the Fe-deficiency response of leaves of wild-type plants. In addition, *erf109* mutant plants accumulated higher Fe levels than the wild-type and constitutively induced several Fe deficiency response genes in shoots, suggesting that *ERF109* is a key node in the regulation of Fe-responsive genes in above-ground plant parts.

## Materials and Methods

### Plant Growth Conditions

The *Arabidopsis thaliana* Col-0 ecotype and *erf109* knock-out mutant lines (SALK_150614) were obtained from the Arabidopsis Biological Resource Center. Seeding holder was made by punching 96 holes on a foam board and filled by the hydroponic solution which solidified with 1% Agargel (A3301, Sigma, MO, United States) containing essential nutrients as described (Rodríguez-Celma et al., 2013), which included 5 mM KNO_3_, 2 mM MgSO_4_, 2 mM Ca(NO_3_)_2_, 2.5 mM KH_2_PO_4_, 70 μM H_3_BO_3_, 14 μM MnCl_2_, 1 μM ZnSO_4_, 0.5 μM CuSO_4_, 10 μM NaCl, 0.2 μM Na_2_MoO_4_, 40 μM FeEDTA, 4.7 mM MES, with pH 5.7. Then, the seeding holders were floated on the hydroponic solution inside boxes. Seeds were stratified on seeding holders at 4°C in the dark for 1 day and subsequently transferred to acclimatize under continuous light (90 μmol m^–2^ s^–1^) and were grown at 21°C in a growth chamber. The hydroponic solution was renewed every 3 days. Plants were transferred to Fe-sufficient or Fe-deficient (−Fe, 0 μM Fe^2+^ supplemented with 100 μM 3-(2-pyridyl)-5,6diphenyl-1,2,4-triazine sulfonate to chelate trace iron) hydroponic solution for treatment. Shoot tissue was harvested for all experiments.

For RNA-seq and quantitative RT-PCR (RT-qPCR) analysis, Col-0 and *erf109* plants were grown in hydroponic solution for 10 days and transferred to Fe-sufficient or Fe-deficient hydroponic solution for an additional 3 days. Three replications for each treatment and genotype were applied for data collection. For Fe concentration analysis, plants were grown in hydroponic solution for 18 days under continuous light and transferred to Fe-sufficient or Fe-deficient solution under continuous light for 3 days. Two replications for each treatment were applied for data collection. For chlorophyll fluorescence and chlorophyll content measurements, plants were grown in Fe-sufficient hydroponic solution for 14 days and transferred to Fe-sufficient or Fe-deficient solution under continuous light for 3 days. Three replications for each treatment were applied for data collection.

### RNA-seq Analysis

For RNA-seq, RNA extraction, sequencing, and annotation were conducted as described (Rodríguez-Celma et al., 2013). Total RNA was extracted from shoots of Col-0 and *erf109* plants using the RNeasy Plant Mini Kit (Qiagen), following the manufacturer’s instructions. The cDNA libraries were constructed with equal amounts of total RNA following the manufacturer’s protocol (Illumina, CA, United States), enriched by PCR amplification and subjected to paired-end sequencing on an Illumina Genome Analyzer II. Data collection was conducted as previously described (Mortazavi et al., 2008). Reads from RNA-seq were mapped to the *Arabidopsis* genome version TAIR10 by Bowtie2 (Langmead and Salzberg, 2012), other unmappable reads were mapped to *Arabidopsis* genome version TAIR10 using BLAT (Kent, 2002). Read counts were calculated and normalized by means of RackJ package^1^ and the TMM-quantile method (Robinson and Oshlack, 2010), respectively. Normalized read counts were further converted into RPKM (Reads Per Kilobase Million) values.

### Differential Gene Expression

To obtain a comprehensive catalog of differentially expressed genes (DEGs), three subsets of DEGs were selected based on mean RPKM compared to control with a *Z*-test *P*-value < 0.05: “Col-Fe” indicates genes that were differentially expressed between Fe-deficient and Fe-sufficient Col-0 plants, “*erf109*-Fe” indicates genes that were differentially expressed between Fe-deficient and Fe-sufficient *erf109* mutant plants, and “*erf109*-regulon” indicates genes that were differentially expressed between *erf109* and Col-0 plants under Fe-sufficient conditions.

### Co-expression Network Construction and Visualization

For DEG clustering, the DEGs of ‘‘Col-Fe,’’ ‘‘*erf109*-Fe,’’ and ‘‘*erf109*-regulon’’ were used as input for the MACCU software^2^ (Lin et al., 2011) to build co-expression networks. The network was based on co-expression relationships with a Pearson’s coefficient greater than or equal to 0.8. The network was visualized using Cytoscape software ver. 3.7.2.^3^ The color was set to show up-regulated (red) and down-regulated (blue) DEGs from log2 fold-change ranging from 2 to −2.

### GO Enrichment and Visualization

Differentially expressed genes with the same regulation pattern in “Col-Fe” and in the “*erf109*-regulon” were selected for functional analysis using the singular enrichment analysis tool in agriGO (Tian et al., 2017), which was applied with the default parameters for *A. thaliana*. The biological process result was visualized using the REVIGO (Supek et al., 2011) tool with the default parameters for *A. thaliana* GO terms. All the DEGs of “Col-Fe,” “*erf109*-Fe,” and “*erf109*-regulon” were put into the MapMan software (Thimm et al., 2004) with log2 fold-change values for visualizing functional categories. The “Ath_AGI_TAIR9_Jan2010” database in MapMan was applied for mapping. The heatmaps were plotted in R (version 4.1.0).

### RNA Expression Analysis

Total RNA was extracted with TRIzol™ Reagent (Invitrogen, MA, United States) following the manufacturer’s instructions. The HiScript II 1st Strand cDNA Synthesis Kit (Bionovas, Toronto, ON, Canada) was used for cDNA preparation. RT-qPCR was performed on the CFX Connect Real-Time PCR Detection System (Bio-Rad, CA, United States) using iQ™ SYBR ^®^ Green Supermix (Bio-Rad, CA, United States). For each reaction, 0.3 μM of the forward and the reverse primers and 100 ng of cDNA template were added. All primers used in this study are listed in Supplementary Table 3. The relative gene expression level was determined according to the 2^–ΔΔCt^ method (Livak and Schmittgen, 2001) and fold-changes were calculated relative to Col-0 values. For each sample, the mRNA abundances of the target genes were normalized to those of the *UBIQTIN10* (At4g05320) gene. Three biological replicates were run for each gene. The final figures were plotted in R (version 4.1.0).

### Iron Concentration

Shoots were rinsed with 1% HCl and at least three times with deionized water, dried at 100°C for 1 h, and kept at 70°C for 3 days. NIST SRM 1573a (tomato leaves) served as a standard. Dried shoots were weighed into a Teflon vessel and digested with 5 ml of 65% HNO_3_ (Merck, Darmstadt, Germany) and 2 ml H_2_O_2_ (Choneye Pure Chemicals, Taipei, Taiwan) in a MarsXpress microwave digestion system (MARS 5 Xpress; CEM). ICP-optical emission spectrometry (ICP-OES; PerkinElmer, MA, United States) was used for metal element analyses. Determination of metal concentration was conducted as described (Shanmugam et al., 2011). The final figures were plotted in R (version 4.1.0).

### Chlorophyll Fluorescence and Chlorophyll Content Measurements

The plant material was acclimated in the dark for 20 min before the measurements. Chlorophyll fluorescence was determined at room temperature using a PAM-2100 (Heinz Walz GmbH, Effeltrich, Germany) fluorometer. Minimal (Fo) and maximal (Sies and Menck, 1992) fluorescence yields of dark-adapted samples were recorded after applying a saturating pulse of light. Maximal quantum yield of photosystem II (Fv/Fm) was calculated from the given Fo and Fm-values using the equation: Fv:Fm = (Fm−Fo):Fm. The effective quantum yield of photosystem II (yield) was calculated according to the equation: Y = (Fm′−Ft): Fm′ = ΔF: Fm′. A Soil Plant Analysis Development (SPAD) chlorophyll meter (Spectrum Technologies, Inc., IL, United States) was applied to measure the chlorophyll content in each line. The SPAD value represents the index of relative chlorophyll content (Richardson et al., 2002). The final figures were plotted in R (version 4.1.0).

### Statistical Analysis

Data were analyzed by applying a Student’s *t*-test with a *P*-value < 0.05.

## Results

### *erf109* Mutants Show Increased Leave Iron Content and Reduced Photosynthesis

**FIGURE 1:**
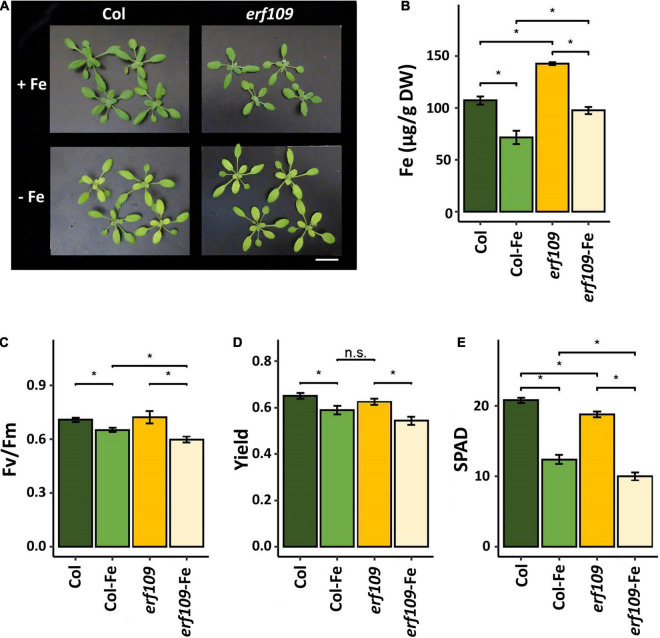
Phenotype of *erf109* mutant plants and Col-0 wild-type. **(A)** Growth (21-day-old plants), **(B)** Fe concentration, **(C)** Fv/Fm ratio, **(D)** quantum yields of PSI, and **(E)** SPAD value of chlorophyll content of Col-0 and *erf109* under Fe sufficiency (Col, *erf109*) or Fe deficiency (Col-Fe, *erf109*-Fe). Statistically significant difference was conducted by Student’s *t*-test at *P* < 0.05 between either two selected treatment or genotype, marked as asterisk. n.s., no significant; +Fe, iron sufficient condition; –Fe, iron-deficiency treatment. Bar, 5 cm.

To explore the effects of *ERF109* on the Fe content and light responsiveness in leaves, we first collected phenotypic data of *erf109* mutant plants. After 72 h of Fe deficiency, no visible differences between the mutant and its wild-type were observed (Figure 1A). However, the Fe concentration of both Col-0 and *erf109* plants was decreased in response to Fe deficiency (Figure 1B). Notably, *erf109* mutant plants exhibited significantly higher Fe levels than the wild-type under both growth conditions (Figure 1B). The maximum quantum yield of photosystem II (Fv/Fm) was reduced upon Fe-deficiency in both Col-0 and *erf109* plants, with *erf109* plants exhibiting significantly lower values than Col-0 under Fe-deficiency (Figure 1C). A similar pattern was observed for the quantum yield of PSI and yield, the difference between the *erf109* and Fe-deficiency Col-0 was, however, not statistically significant (Figure 1D). SPAD values, providing a read-out for the chlorophyll content, were reduced in both genotypes under Fe-deficient conditions, with significantly lower values in *erf109* plants under Fe-sufficient and Fe-deficient conditions (Figure 1E). Together, these results indicate that in *erf109* plants the chlorophyll content and photosynthetic efficiency was decreased relative to the wild-type despite higher Fe concentrations in the leaves even under Fe-sufficient conditions, suggesting that the additional Fe may be not physiologically available.

### Transcriptional Changes Caused by Dysfunctional *ERF109* Mimics Iron Deficiency

**FIGURE 2:**
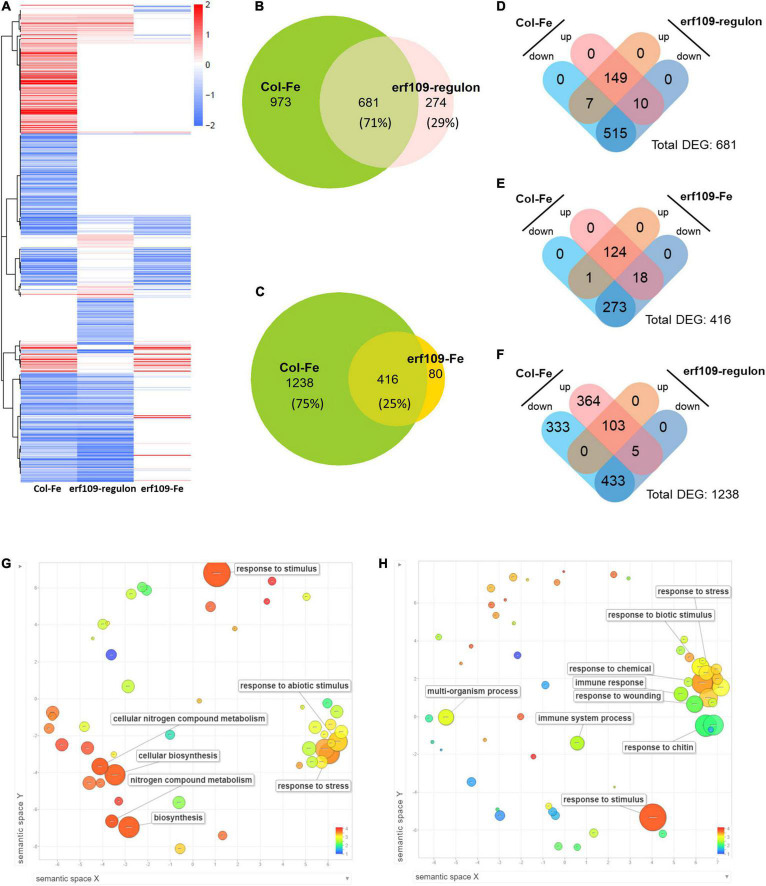
Differentially expressed transcripts, expression profiles and gene ontologies of Col-Fe, the *erf109*-regulon and *erf109*-Fe. **(A)** Heatmap of three transcriptomic profiles. Red and blue color indicates up- and down-regulated expression of DEGs with log2-fold change, respectively. **(B)** Venn diagram of DEGs from Col-Fe and the *erf109*-regulon. **(C)** Venn diagram of DEGs from Col-Fe and *erf109*-Fe. Distribution of up- and down-regulated DEGs overlapping between Col-Fe and the *erf109*-regulon **(D)**, overlapping between Col-Fe and *erf109*-Fe **(E)**, and *erf109*-regulon DEGs in specific of 1,238 Col-Fe DEGs **(F)**. GO analysis (biological process) of 149 up- **(G)** and 515 down-regulated DEGs **(H)** overlapping in the Col-Fe and *erf109*-regulon data sets. Dot size indicates –log10 (*P-*value), the log value is labeled with the color bar from 1 to 4.

To uncover a possible role of *ERF109* in the Fe deficiency response of Arabidopsis shoots, we subjected wild-type plants and *erf109* mutant lines to transcriptional profiling using RNA-seq technology. Genes that were differentially expressed between the mutant and its wild-type are here referred to as the *erf109*-regulon, while Fe-responsive genes of the wild-type and mutant plants were designated Col-Fe and *erf109-*Fe, respectively. To explore the global regulation pattern of the three DEG subsets, heatmaps of log fold-change values were generated (Figure 2A). Conspicuously, highly similar expression patterns were observed for Col-Fe and the *erf109*-regulon. A subset of 681 of the total 955 DEGs in the *erf109*-regulon overlapped with the Col-Fe DEGs (Figure 2B), with 149 up-regulated and 515 down-regulated genes in both Col-Fe and the *erf109*-regulon (Figure 2D). The significant overlap and the similar direction of gene regulation in Fe-deficient wild-type plants and *erf109* mutants indicate a partial constitutive Fe deficiency response in shoots of *erf109* mutant plants. Under Fe-deficient conditions, a subset of 416 out of 1,654 DEGs that responded to Fe starvation in the wild-type were also responsive to the Fe regime in *erf109* mutants; the majority of Col-Fe genes (1,238 DEGs), however, were not affected in the mutant (Figure 2C). The subset of overlapping DEGs revealed a similar direction of expression in both Col-Fe and *erf109-*Fe, with 124 genes that were induced and 273 genes that were repressed under Fe starvation (Figure 2E). From the 1,238 Col-0-specific Fe-responsive DEGs that were not affected in *erf109* mutant plants under Fe deficiency, 103 induced and 433 repressed genes were regulated similarly in the *erf109*-regulon but lost their differential expression in the *erf109*-Fe dataset (Figure 2F). The function of the 681 DEGs that were comprised in both the Col-Fe subset and the *erf-109* regulon were further analyzed by their gene ontology. Among these genes, 149 up-regulated DEGs are involved in the response to abiotic stimulus and cellular nitrogen compound metabolism (Figure 2G), and 515 down-regulated DEGs are associated with the response to biotic stimulus, wounding, chitin, and the immune system (Figure 2H). These results indicate that knock-out of *ERF109* induces gene expression patterns that were to a large part similar to those observed in Fe-deficient wild-type plants and predicted to be involved in establishing immunity.

**FIGURE 3:**
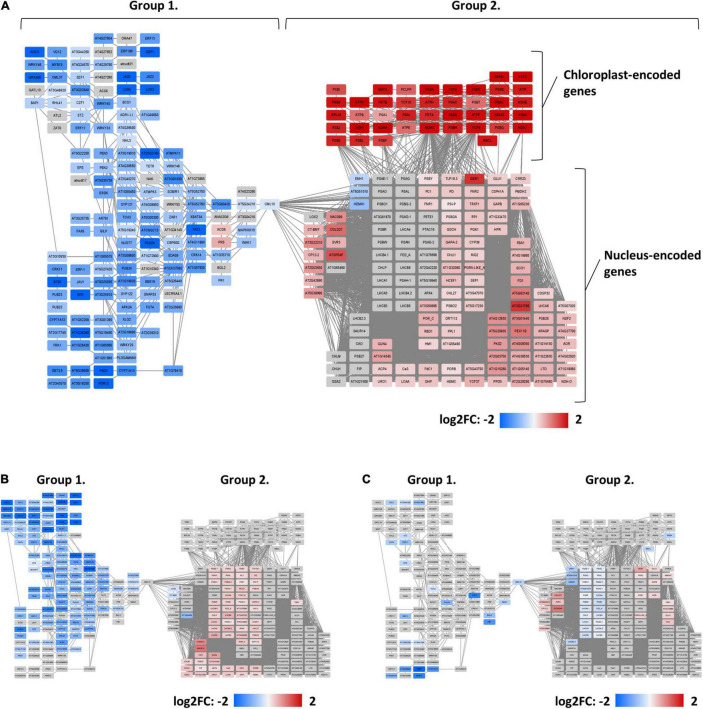
Co-expression networks of genes that were differentially expressed between genotypes or treatments. **(A)** Col-Fe, **(B)** *erf109*-regulon, and **(C)** *erf109-Fe*. Log2-fold change of DEGs are labeled by color: gray, no significant changes; red, up-regulated; blue, down-regulated.

To further explore the changes in the transcriptome induced by either Fe starvation, dysfunctional *ERF109*, or both conditions, a co-expression network was constructed for the gene subsets Col-Fe, *erf109-*Fe, and *erf109*-regulon. Total DEGs were divided into two main clusters by their co-expression relationships: group 1 comprises mainly down-regulated genes, while group 2 comprises mostly up-regulated genes, which can be further subdivided into chloroplast-encoded and nucleus-encoded genes (Figure 3A). In the Col-Fe network, group 1 genes are connected to group 2 DEGs, which in turn is connected to a group of chloroplast-encoded DEGs which were significantly up-regulated (Figure 3A). The latter subset is not significantly regulated within the *erf109*-regulon (Figure 3B). Under Fe-deficient conditions, genes in *erf109*-Fe lost most of the response within both groups; some group 2 DEGs were even down-regulated (Figure 3C). Taken together, the network revealed that the overlap of the *erf109*-regulon and Col-Fe partly comprises the down-regulated genes in group 1, and the *erf109*-regulon completely abolishes the up-regulation of chloroplast-encoded genes. Moreover, defective *ERF109* dramatically affected the gene expression pattern observed under Fe-deficient conditions in wild-type plants.

### *ERF109* Affects the Immune Response and Photosynthesis Under Iron-Deficient Conditions

**FIGURE 4:**
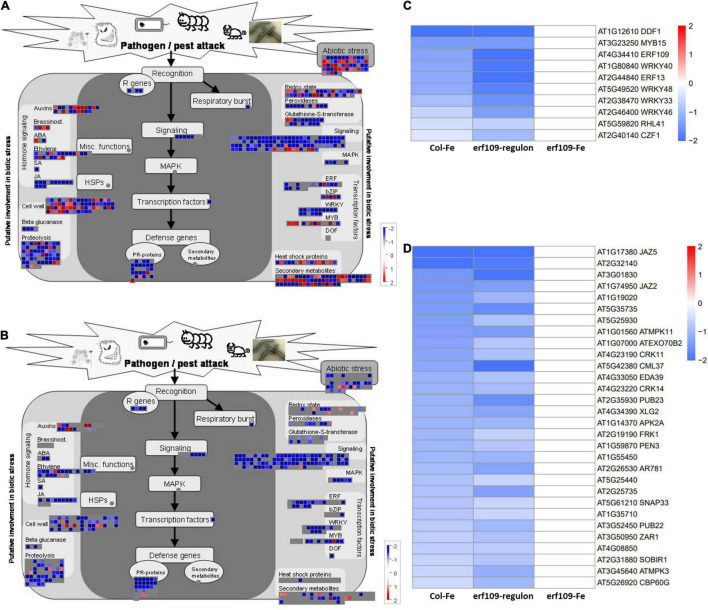
Functional visualization of DEGs involved in pathogen defense. **(A)** Col-Fe, **(B)** *erf109*-regulon. Graphs were generated with MapMan software (Thimm et al., 2004). Heatmap depicting log2 fold-changes of the transcription factors **(C)** and other functional DEGs **(D)** in group 1. Log2-fold changes of DEGs are labeled by color: white, no significant changes; red, up-regulated, blue: down-regulated.

To further investigate the function of the DEGs in the *erf109*-regulon, a gene ontology analysis using the MapMan software was employed. The analysis revealed that the most significant down-regulated DEGs are involved in biotic and abiotic stress responses, including R genes that code for proteins recognizing specific pathogen effectors, induction of the respiratory burst, signaling, and defense genes such as PR-proteins. Genes putatively involved in processes related to biotic stress such as hormone signaling, cell wall, proteolysis, and transcription factors were significantly reduced in both Col-Fe and *erf109*-regulon (Figures 4A,B). These defense-related DEGs comprise transcription factors such as *DWARF AND DELAYED FLOWERING* (*DDF*), SG2-type R2R3-MYB (*MYB*), *WRKY*, a putative zinc-finger (*RHL*), and other ERF family proteins (Figure 4C) as well as signaling proteins such as calmodulin-like proteins (*CML*), MAP kinases (*MPK*), and other defense-related proteins including a cysteine-rich receptor-like protein kinase (*CRK*), a *PLANT U-BOX* (*PUB*) protein, *PROTEIN KINASE 2A* (*APK2A*), and *SUPPRESSOR OF BIR1* (*SOBIR1*) (Figure 4D). The observation that these stress response-associated genes were commonly affected in Col-Fe and in the *erf109*-regulon suggests that *ERF109* governs the regulation of Fe-responsive immunity-associated genes that are part of the Fe deficiency response.

**FIGURE 5:**
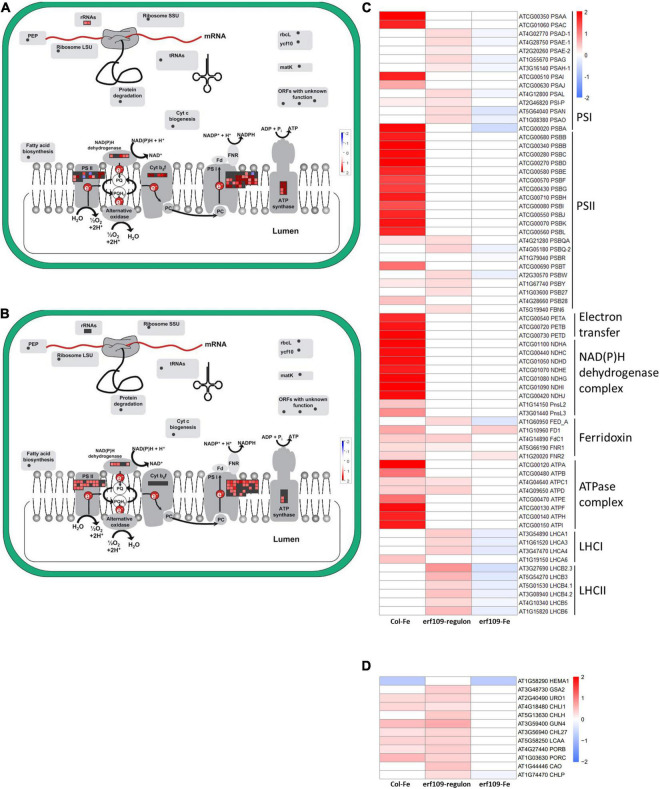
Differentially expressed genes encoding photosynthesis-related proteins. **(A)** Col-Fe, **(B)** *erf109*-regulon. Graphs were generated with MapMan software (Thimm et al., 2004). Heatmap depicting log2 fold-changes of genes encoding photosynthesis subunits **(C)** and genes involved in chlorophyll biosynthesis **(D)** in group 2. Log2-fold changes of DEGs are labeled by color: white, no significant changes; red, up-regulated; blue, down-regulated.

In addition, induction of genes encoding photosystem subunits was observed in both Col-Fe and in the *erf109*-regulon, including a large fraction of genes encoding proteins involved in photosynthetic electron transport which showed increased transcription in Col-Fe and, to a lesser extent and partly different from those of Col-Fe, in *erf109* plants (Figures 5A,B). In Col-Fe, the most highly expressed genes were encoded by plastidic DNA, including genes encoding proteins associated with the reaction centers of PSI and PSII, electron transfer proteins, NAD(P)H dehydrogenases, and ATP synthases (Figure 5C). By contrast, in the *erf109*-regulon, other genes associated with PSI and PSII, ferredoxin, and light-harvesting chlorophyll *a*/*b*-binding proteins were higher expressed than in the wild-type. When *erf109* plants were subjected to Fe-deficient conditions, genes involved in these processes were rather repressed, showing a pattern that was clearly distinct from the other two transcriptomes (Figure 5C).

Chlorophyll plays an essential role in light harvesting and electron transfer in photosynthesis (Schreiber et al., 1995). The expression of genes encoding enzymes in tetrapyrrole biosynthesis such as *HEME2*, *GUN4*, *CHLI1*, and *PORB* was enhanced in both Col-Fe and in the *erf109*-regulon, but this induction was abolished in Fe-deficient *erf109* plants (Figure 5D). A comparison between the genotypes suggests that *ERF109* is mainly required for mounting defense responses under conditions of Fe deficiency. In addition, defects in *ERF109* under Fe-deficiency is accompanied by partial repression of photosynthesis subunits and genes involved in chlorophyll biosynthesis.

### Mutations in *ERF109* Increased the Expression of Iron-Responsive Genes

**FIGURE 6:**
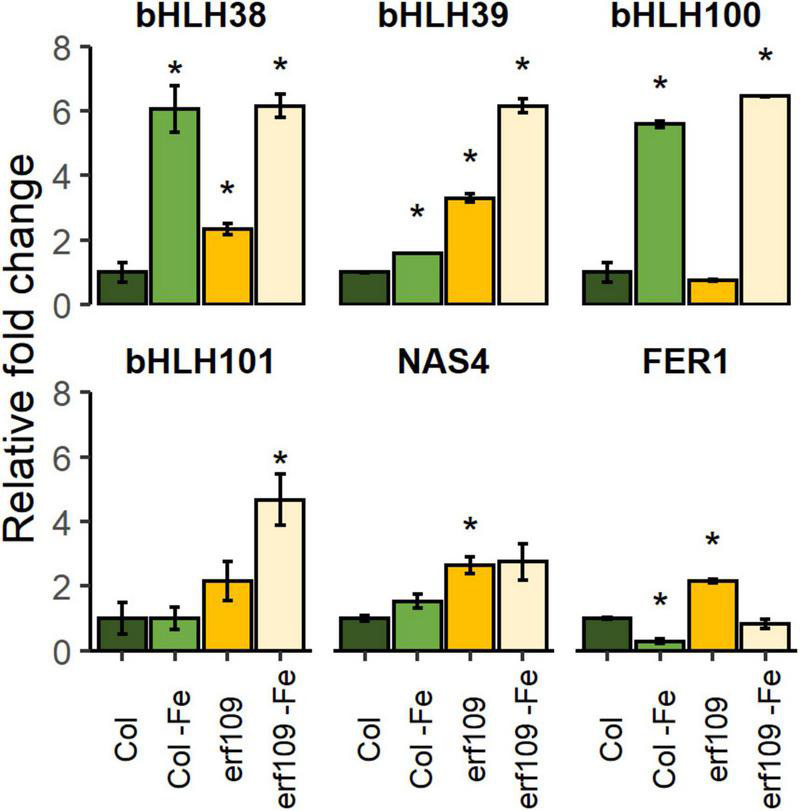
Quantitative RT-PCR analysis of relative fold change of Fe-responsive genes in Col-0 and *erf109* plants under Fe-sufficient or Fe-deficient conditions. The level of transcript was normalized to Col-0 under Fe-sufficient conditions. Data are presented as mean ± SE from two biological replicates. Student’s *t*-test significantly different at *P* < 0.05 is marked with an asterisk.

To investigate how *ERF109* influences cellular Fe homeostasis in leaves, RT-qPCR of a subset of Fe-responsive genes was selected, including four clade Ib bHLH proteins (*bHLH38/39/100/101*) that are critically required for the induction of all Fe deficiency responses (Wang et al., 2013), a gene encoding the Fe chelator nicotianamine (*NICOTIANAMINE SYNTHASE4*, *NAS4*) that is involved in metal translocation within the plant (Nozoye, 2018), and Fe storage protein *FERRITIN1* (*FER1*) (Harrison and Arosio, 1996). The expression of *bHLH38/39/101* was significantly increased in *erf109* mutant plants and even more so after the Fe deficiency treatment (Figure 6). The expression of *NAS4* and *FER1* was higher in *erf109* than in Col-0 plants in both Fe-sufficient and Fe-deficient conditions (Figure 6). Mutations in *ERF109* increased *bHLH38/39/101* and *NAS4* expression, mimicking Fe deficiency. While *FER1* was down-regulated in Fe-deficient Col-0 plants, *FER1* transcript levels were increased in *erf109* mutants under Fe-sufficient conditions, reflecting the higher Fe content in leaves of *erf109* mutants (Figure 6).

### Homozygous *erf109* Mutants Simulate Down-Regulation of Defense Genes and Up-Regulation of Photosynthesis Genes Within the Iron-Deficiency Response

**FIGURE 7:**
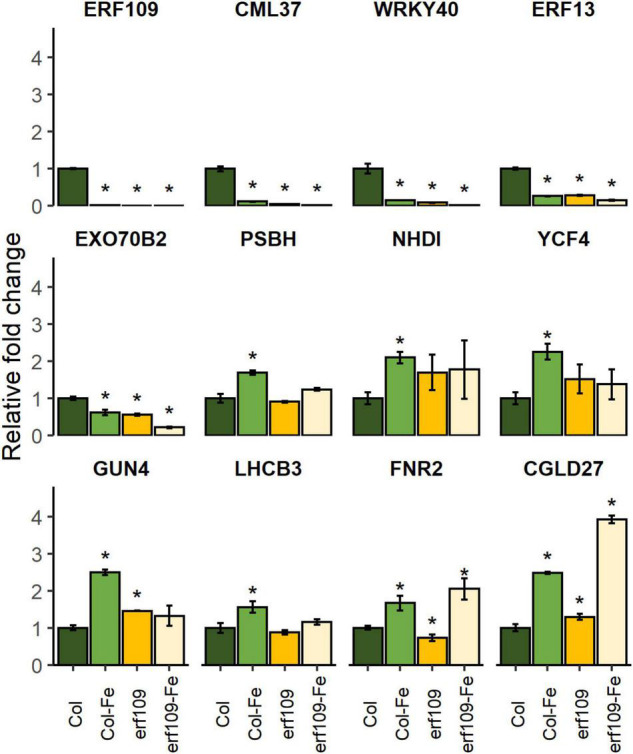
Quantitative RT-PCR analysis of relative fold change of DEGs in Col-0 and *erf109* under Fe-sufficient (Col, *erf109*) or Fe-deficient conditions (Col-Fe, *erf109*-Fe). The level of transcript was normalized to Col-0 under Fe-sufficient conditions. Data are presented as mean ± SE from three biological replicates. Student’s *t*-test significantly different at *P* < 0.05 is marked with an asterisk.

To validate the differential expression pattern observed in the RNA-seq analysis, RT-qPCR of DEGs selected from the groups of defense-related, photosynthesis-related, and chloroplast-encoded genes was carried out. The criterion for the selection of these DEGs was based on the significance of the fold-changes and the molecular function of the genes. In wild-type plants, *ERF109* was down-regulated in response to Fe-deficiency treatment; no *ERF109* transcripts could be detected in the knock-out lines (Figure 7). Decreased expression of the calmodulin-like protein *CML37*, transcription factors involved in the defense response, in negotiating between abscisic acid (ABA) and ethylene (*WRKY40*, *ERF13*), and the exocyst subunit EXO70 family protein B2 (*EXO70B2*) was observed in Fe-deficient Col-0 plants and in *erf109* mutant plants under both Fe-sufficiency and Fe-deficiency (Figure 7). By contrast, the expression of the ferredoxin-NADP reductase *FNR2*, photosystem II light-harvesting protein *LHCB3*, and the chlorophyll biosynthesis gene *GUN4* was significantly increased upon Fe-deficiency in Col-0 but not in *erf109* mutant plants (Figure 7). *CONSERVED IN THE GREEN LINEAGE AND DIATOMS 27* (*CGLD27*) was up-regulated in both Fe-deficient Col-0 and *erf109* plants (Figure 7). However, chloroplast-encoded genes such as the subunit of the chloroplast NAD(P)H dehydrogenase complex *NDHI*, photosystem II reaction center protein *PSBH*, and a protein required for photosynthesis I assembly and stability (*YCF4*) were only responsive to Fe deficiency in Col-0 plants (Figure 7). All expression patterns were in accordance with the RNA-seq data.

## Discussion

Chlorosis on the young leaves is the most prominent symptom caused by Fe limitation. The chlorophyll content and the maximum quantum yield of photosystem II (Fv/Fm) serve as a phenotypic index of Fe deficiency in a variety of studies (Bertamini et al., 2002; Timperio et al., 2007; Kobayashi et al., 2013; Lei et al., 2014; Liu et al., 2017a; Rajniak et al., 2018; Becker et al., 2020; Tombuloglu et al., 2020). Upon Fe deficiency, wild-type plants showed a significant reduction in Fe levels, chlorophyll concentration, Fv/Fm, and quantum yield of PSI. Homozygous *erf109* lines, on the other hand, exhibited elevated levels of Fe under both Fe-sufficient and Fe-deficient conditions when compared with Col-0. In addition, the *erf109*-regulon revealed a pattern of gene expression similar to that of Fe-deficient wild-type plants in leaves, suggesting that – independent of the external Fe supply – *erf109* plants exhibit a constitutive Fe-deficiency response. One of the potential reasons for the pronounced chlorosis of *erf109* plants lies in the increment of chlorophyll breakdown. ABA signaling can induce the decomposition of chlorophyll (Kuai et al., 2018; Asad et al., 2019). The expression of *LHCB3* was significantly upregulated in *wrky40* mutants, indicating that ABA positively regulated the expression of light-harvesting chlorophyll *a*/*b*-binding proteins *via* the *WRKY40* transcriptional repressor (Liu et al., 2013). The results of the present study also showed that down-regulation of *WRKY40* is accompanied by up-regulation of *LHCB3* in *erf109* mutant plants (Figure 7). The LHCB3 antenna subunit is a crucial participant in the modulation of PSII antenna size upon long-term acclimation to increased light levels in thylakoids (Albanese et al., 2016). Other research supported the assumption that LHCB family proteins are involved in ABA signaling by modulating ROS homeostasis (Xu et al., 2012). Another study also points out that WRKY40 is required for upregulation of *ERF109* by stress stimuli and H_2_O_2_ (Matsuo et al., 2015). These data indicate that *ERF109* potentially cooperates with hormones and the photosynthesis receptors to regulate immunity *via* WRKY40.

Several ethylene response factors such as *ERF4* and *ERF72* have been shown to exert negative effects on the Fe deficiency response in Arabidopsis (Liu et al., 2017a,b). DEGs in the *erf109* transcriptome related to hormones are At5g35735 (auxin), *ERF13*, and *ERF13/109* (ethylene), *JAZ2/5*, *LOX2/3/4*, and *AOC3* (jasmonic acid; JA) (Supplementary Tables 1, 2). Both *ERF13* and *ERF109* are under the regulation of the JA-activated transcription factors MYC2/MYC3/MYC4 (Van Moerkercke et al., 2019). Jasmonate signaling has been observed in rice roots exposed to 3 h Fe deficiency (Kobayashi et al., 2016), exerting a repressing effect on Fe uptake (Cui et al., 2018). Although *ERF109* was found to be up-regulated in response to a 6 h Fe-deficiency treatment (Hsieh et al., 2022), the present results revealed a repression of the gene after 72 h Fe deficiency in Col-0 leaves (Figure 7). It may thus be assumed that *ERF109* and JA-signaling genes were inversely regulated in the early and later Fe deficiency response. The molecular mechanisms by which *ERF109* and JA signaling interact on the Fe deficiency response remain, however, to be unraveled.

Previously, *ERF109* was shown to serve as a major regulator of the light stress response and ROS homeostasis (Khandelwal et al., 2008; Matsuo et al., 2015; Carmody et al., 2016; Pospisil, 2016; Kong et al., 2018). In the present study, transcriptome and phenotypic data supported these findings and revealed that *ERF109* is particularly important for balancing the Fe status in addition to immunity and the response to light stress. In addition, *ERF109* was severed as a marker gene for high light-induced SAA (Carmody et al., 2016). In the present study, transcriptome analysis revealed that the majority of Fe-deficiency-induced DEGs in leaves were affected by a lack of functional *ERF109.* A co-expression network derived from genes that were differentially expressed between the growth- and genotypes revealed that the overlap of the *erf109*-regulon and Col-Fe consisted of defense-related and photosynthesis-related genes. Moreover, the mutation in *ERF109* caused de-regulation of most Fe-related genes in *erf109* plants under Fe-deficient conditions (Figure 3C). Moreover, Fe levels in *erf109* plants were higher than in the Col-0 line (Figure 1B). From the lack of significant regulation of Fe-responsive genes in the *erf109-Fe* transcriptome and the increased Fe levels in *erf109* mutant plants (Figures 1B, 3C), it may be speculated that the Fe deficiency response is constitutively induced in *erf109* leaves. These results indicate that *ERF109* potentially governs the defense response and, in part, photosynthesis and plays an important role in the Fe-deficiency response. Decreased expression of defense-related genes was the major change in the *erf109*-regulon and Col-Fe transcriptome, supporting the supposition that *ERF109* controls the expression of immune networks in plants. Compromised Fe homeostasis was found to affect the immune system in plants. For instance, Fe aggregation was observed in corn, barley, oat, sorghum, and millet after attack by powdery mildew *Blumeria graminis* f. sp. *tritici* (Bgt) (Liu et al., 2007). Ferric Fe accumulation at the pathogen attack site together with intracellular Fe depletion was shown to promote the transcription of pathogenesis-related genes (Liu et al., 2007). Another report showed that the Fe nutritional status affected infection of maize by *Colletotrichum graminicola* (Ye et al., 2014). These studies revealed a close relationship between Fe homeostasis and the immune system of plants.

The immune system of plants is a complex network that integrates different regulative gene families such as stress-related genes, signaling, transcription factors, and response elements (Ngou et al., 2021). From our transcriptomic survey, it appears that *ERF109* controls different defense-related signaling mechanisms and pathways associated with abiotic and biotic stresses. One of the best described signaling cascades is the Ca^2+^-based response. Ca^2+^ fluxes act as an intracellular secondary messenger, which is initiated by Ca^2+^ sensor proteins such as calmodulin and calmodulin-like proteins (Clapham, 2007; Bootman and Bultynck, 2020). *CALMODULIN-LIKE PROTEIN* (*CML37*) is highly expressed in younger leaves, and quickly (typically within 0.5–3 h) responds to wounding, osmotic stress, and drought (Vanderbeld and Snedden, 2007). It was shown that Arabidopsis lacking functional *CML37* is highly susceptible to drought stress. Moreover, *CML37* is a positive regulator of the plant hormone ABA (Scholz et al., 2015). In the present study, expression of *CML37* was significantly decreased in Col-Fe, *erf109*-regulon, and *erf109*-Fe, suggesting that *ERF109* participates in the regulation of intercellular Ca^2+^ signal transduction in response to Fe deficiency.

The WRKY transcription factor family plays essential roles in pathogen defense (Rushton et al., 2010; Chen et al., 2017). WRKY40 targets several downstream genes involved in the perception and transduction of microbial-associated and damage-associated molecular pattern-triggered immunity, the production of secondary indolic metabolites, and the modulating of distinct plant hormone pathways (Birkenbihl et al., 2017). Previous research revealed that ERF13 protein interacts with ERF109 and WRKY40, and is involved in the defense against herbivory (Chia, 2013; Miyamoto et al., 2019). All three genes are down-regulated in Col-Fe, *erf109*-regulon, and *erf109*-Fe, suggesting that *ERF13*, *WRKY40*, and *ERF109* cooperate in the Fe deficiency response in immune signaling and hormone modulation.

Genes that robustly respond to the Fe regime across different transcriptomic datasets have been referred to as the “ferrome” (Schmidt and Buckhout, 2011; Hsieh et al., 2022). A subset of 12 genes that are within the *erf109*-regulon and the Col-Fe subset belong to the shoot ferrome (Hsieh et al., 2022), among them the ferritin *FER1*, a sensitive marker for the Fe status, the chlorophyll biosynthesis gene *PORB*, and the U-box type E3 ubiquitin ligase *PUB23*. Previous research demonstrated that *PUB22*, *PUB23*, and *PUB24* negatively regulate PAMP-triggered responses (Trujillo et al., 2008). In PAMP-triggered responses, PUB22 targets a subunit of the exocyst complex encoded by *EXO70B2*, which mediates the process of vesicle tethering during exocytosis (Stegmann et al., 2012). *EXO70B2* also regulates the receptor of bacterial flalso re or its immunogenic epitope flg22 at the plasma membrane, inhibiting the infection of bacterial pathogens by influencing the initiation of microbe-associated molecular pattern-triggered immunity (Wang et al., 2020). Decreased expression of *EXO70B2* in Col-Fe, the *erf109*-regulon and *erf109*-Fe suggests compromised defense responses in *erf109* mutant plants. From what has been mentioned above, the decline in expression of *ERF109* and other defense DEGs in response to Fe deficiency suggests a link between Fe deficiency signaling and defense pathways.

The reasons for the increase of the Fe content in *erf109* mutant leaves were investigated through the determination of the expression of six typical Fe deficiency-response genes. Although the four Ib subgroup bHLH proteins show functional redundancy in the Fe deficiency responses, multiple mutant lines of these bHLH genes in Arabidopsis exhibited differential degrees of chlorosis and *IRT1* and *FRO2* gene expression levels (Wang et al., 2013), suggesting that these bHLH proteins play distinct roles in regulating Fe uptake (Wang et al., 2013). All four clade Ib bHLH proteins form heterodimers with FIT to enhance the transcription of Fe uptake transporters in root, but the genes are also induced by Fe deficiency in leaves (Shen et al., 2016). RT-qPCR analysis revealed robust induction of *bHLH38/39* expression in *erf109* leaves under Fe-sufficient conditions (Figure 6), suggesting that *ERF109* is repressing the Fe deficiency response. High Fe levels in the tomato mutant *chloronerva* was found to be caused by compromised biosynthesis of nicotianamine, which resulted in excessive Fe uptake and precipitation of Fe in the form of insoluble ferric phosphate compounds, protecting the cells from Fe overload (Becker et al., 1995). The nicotianamine synthase *NAS4* showed increased expression in *erf109* mutants induction under Fe-sufficient conditions compared to Col-0 wild-type plants (Figure 6), supporting the supposition that *ERF109* is negatively regulating cellular Fe homeostasis. The regulation of *FER1* corresponded to the Fe content in *erf109* lines and Col-0 plants under the various Fe regimes (Figure 6). Together, these data support the hypothesis that compromised expression of *ERF109* perturbs Fe homeostasis at an early stage of the signaling cascade by repressing the Fe acquisition machinery.

## Conclusion

To sum up, the highly similar transcriptome expression patterns of Fe-sufficient *erf109* mutants and Fe-deficient wild-type plants suggest that *ERF109* is an upstream regulator of the Fe deficiency-induced immunity response. Moreover, higher Fe levels in *erf109* leaves and reduced photosynthetic efficiency indicate that *ERF109* attunes the physiologically available Fe for photosynthesis. We uncovered that the immunity network induced by Fe deficiency is the major target of ERF109 in shoots, which included down-regulation of *CML37*, *WRKY40*, *ERF13*, and *EXO70B2*. Moreover, dysfunctional *ERF109* reduced the photosynthetic efficiency under Fe-deficient conditions. We provided evidence for a regulatory role of *ERF109* in plant Fe homeostasis and set the stage for future studies and crop breeding to generate germplasms with improved systemic immunity and Fe uptake efficiency.

## Data Availability Statement

The datasets presented in this study can be found in online repositories. The names of the repository/repositories and accession number(s) can be found below: National Center for Biotechnology Information (NCBI) BioProject database under accession number PRJNA793283.

## Author Contributions

C-LY wrote the manuscript and analyzed the data. Y-TH carried out the experiments. WS participated in experimental design and manuscript writing. PK participated in data analysis. M-TC conceived the original idea. I-CP contributed to experimental design, project management, and coordination and drafted the manuscript. All authors read and approved the final manuscript.

## Conflict of Interest

The authors declare that the research was conducted in the absence of any commercial or financial relationships that could be construed as a potential conflict of interest.

## Publisher’s Note

All claims expressed in this article are solely those of the authors and do not necessarily represent those of their affiliated organizations, or those of the publisher, the editors and the reviewers. Any product that may be evaluated in this article, or claim that may be made by its manufacturer, is not guaranteed or endorsed by the publisher.

## Footnotes

1
http://rackj.sourceforge.net/

2
http://maccu.sourceforge.net/

3
https://cytoscape.org/
